# Regeneration and functional recovery of the completely transected optic nerve in adult rats by CNTF-chitosan

**DOI:** 10.1038/s41392-022-01289-0

**Published:** 2023-02-27

**Authors:** Xiao Liu, Fei Hao, Peng Hao, Jingxue Zhang, Liqiang Wang, Si-Wei You, Ningli Wang, Zhaoyang Yang, Kwok-Fai So, Xiaoguang Li

**Affiliations:** 1 Department of Neurobiology, School of Basic Medical Sciences, Capital Medical University, 100069 Beijing, China; 2 School of Engineering Medicine, Beijing Key Laboratory for Biomaterials and Neural Regeneration, Beihang University, 100083 Beijing, China; 3 Beijing Tongren Eye Center, Beijing Tongren Hospital, Capital Medical University, Beijing Ophthalmology and Visual Sciences Key Laboratory, 100005 Beijing, China; 4 Department of Ophthalmology, The Third Medical Center, Chinese PLA General Hospital, 100089 Beijing, China; 5 Department of Ophthalmology, Xijing Hospital, The Fourth Military Medical University, 710032 Xi’an, Shanxi Province China; 6 Guangdong-Hongkong-Macau Institute of CNS Regeneration, Ministry of Education CNS Regeneration Collaborative Joint Laboratory, Jinan University, 510632 Guangzhou, Guangdong Province China; 7 Bioland Laboratory (Guangzhou Regenerative Medicine and Health Guangdong Laboratory), 510530 Guangzhou, Guangdong Province China; 8 Department of Ophthalmology and State Key Laboratory of Brain and Cognitive Sciences, The University of Hong Kong, 999077 Hong Kong, China; 9 Center for Brain Science and Brain-Inspired Intelligence, Guangdong-Hong Kong-Macao Greater Bay Area, 510515 Guangzhou, Guangdong Province China; 10 Co-innovation Center of Neuroregeneration, Nantong University, 226001 Nantong, Jiangsu Province China; 11 Beijing International Cooperation Bases for Science and Technology on Biomaterials and Neural Regeneration, Beijing Advanced Innovation Center for Big Data-Based Precision Medicine, Beihang University, 100083 Beijing, China

**Keywords:** Regeneration and repair in the nervous system, Diseases of the nervous system

**Dear Editor**,

The optic nerve, which belongs to the central nervous system (CNS), cannot regenerate when injured in adult mammals.^1^ Up to now, no readily translatable measures are available for repairing a severely injured optic nerve. Herein we demonstrated that ciliary neurotrophic factor (CNTF)-chitosan enabled the reconstruction and functional recovery of the adult rat visual system, thus shedding light on the clinical potential for repairing the severely injured optic nerve.

In this study, we first examined the release kinetics of CNTF from CNTF-chitosan. At the physiological temperature, CNTF-chitosan could release CNTF continuously for 12 weeks (Supplementary Fig. S1d). Next, we investigated the effect of CNTF-chitosan on axonal regeneration of retinal ganglion cells (RGCs). Seven weeks after optic nerve transection (removal of a 1-mm-long optic nerve, Fig. 1a, Supplementary Fig. S1a–c), we injected cholera toxin subunit-B (CTB) conjugated to Alexa-Fluor-555 into the vitreous of the eye to trace the regenerated RGC axons. In the empty tube group, very few CTB^+^ axons sprouted for short-distance from the proximal optic nerve stump with disordered extension trajectories, no CTB^+^ axons were found in the middle part of the lesion site and the distal optic nerve stump (Supplementary Fig. S2, Fig. 1e). However, in the CNTF-chitosan group, plenty of CTB^+^ axons grew out of the proximal optic nerve stump, with obvious directionality, and extended distally in a cone shape to form a nerve bundle (Fig. 1b’, e). Scattered CTB^+^ axons were seen around the undegraded CNTF-chitosan at the lesion site in different shapes, such as axon bifurcation, axonal turn-back, and ring formation (Fig. 1b, Supplementary Fig. S3). The regenerated RGC axons not only passed across the lesion site but also grew into the distal degenerate optic nerve. When the regenerated axons grew into the distal optic nerve stump, most axons terminated growth soon afterwards (Supplementary Fig. S3-V). Interestingly, we found that a large number of CTB^+^ axons formed a nerve bundle and grew inwards from the lateral side of the distal optic nerve (Fig. 1b”-IV–VI, e). The regeneration of RGC axons was accompanied by myelination. Transmission electron microscopy showed that, in the CNTF-chitosan group, a typical small nerve bundle was found in the middle part of the lesion site, exhibiting different levels of axonal myelination (Supplementary Fig. S4a). Around the distal stump, multiple nerve bundles formed by the myelinated axons were seen divorced from the degenerate optic nerve (Supplementary Fig. S4b).

**Fig. 1:**
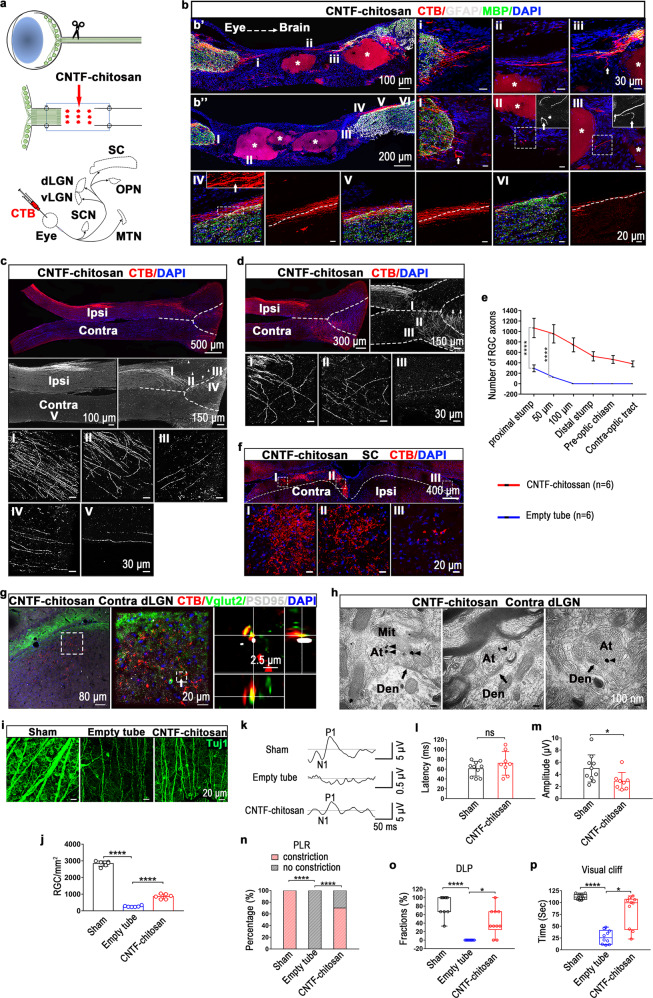
CNTF-chitosan enables the reconstruction and functional recovery of the adult rat visual system. **a** Schematics of optic nerve transection, CNTF-chitosan implantation and CTB tracing. **b** CTB-labeled RGC axons from the CNTF-chitosan group. High-magnification images of the marked regions are shown in b’(i–iii) and b”(I–VI). The arrows in b’(iii) and b”(III) point to axonal turn-backs. The arrows in b”(II) and b”(IV) indicate axon bifurcations. The arrow in b”(I) indicates a ring formation. The white dashed lines indicate the boundary of the optic nerve. The white asterisks indicate CNTF-chitosan. **c**, **d** CTB-labeled RGC axons pass through the optic chiasm. The black and white images are the decolorization images of the CTB layer. High-magnification images of the marked regions are shown in c (I–V) and d (I–III). The arrowheads in (**c**) indicate the axons in the ipsilateral optic tract, and the arrows in (**d**) indicate the axons in the ventral hypothalamus. The white dotted lines indicate the midline of the optic chiasm, the boundaries of the optic nerve and the optic tract. **e** Number of the regenerated axons as a function of distance from the proximal stump of the optic nerve (*n* = 6, mean ± SD, Proximal stump: ****P < 0.0001; 50 um: ****P < 0.0001, unpaired Two-tailed Student’s t-test with Welch’s correction). **f** CTB-labeled axons in the SC. High-magnification images of the marked regions are shown in (I–III). **g** CTB-labeled RGC axons regenerate to the dorsal LGN (dLGN). The Z-stacks of the white arrowed area show that CTB^+^ axons are co-labeled with the presynaptic marker (Vglut2) and closely adjacent to the postsynaptic density (PSD95). **h** Immunoelectron microscopy shows synaptic reorganization in the dLGN. The arrows show the synaptic structure, and the arrowheads show the nanogold particles. At axonal terminal, Den dendrite, Mit, mitochondria. **i**, **j** RGC survival immune-stained with Tuj1 (*n* = 6, mean ± SD, Sham vs. Empty tube, ****P < 0.0001; Empty tube vs. CNTF-chitosan, ****P < 0.0001; one-way ANOVA, LSD). **k**–**m** F-VEP. The average latency of P1 wave (Sham control *n* = 10, CNTF-chitosan *n* = 8, mean ± SD, *P* = 0.2505, unpaired Two-tailed Student’s t-test); ns not significant statistically. The average amplitude of N1-P1 wave (Sham control *n* = 10, CNTF-chitosan *n* = 8, mean ± SD, *P = 0.0353, unpaired Two-tailed Student’s t-test). **n** PLR. The ratio of PLR in the injured eyes (*n* = 10, Sham vs. Empty tube, ****P < 0.0001, Two-sided Fisher’s exact test; Empty tube vs. CNTF-chitosan, ****P < 0.0001, Two-sided Fisher’s exact test). **o** DLP. The rate of rats completing the tests (*n* = 10, Sham vs. Empty tube, ****P < 0.0001, Empty tube vs. CNTF-chitosan, *P = 0.0103, Kruskal-Wallis, Dunn’s). **p** visual cliff. The time of the rats staying at the shallow end (*n* = 10, Sham vs. Empty tube, ****P < 0.0001, Empty tube vs. CNTF-chitosan, *P = 0.0194, Kruskal-Wallis, Dunn’s). Ipsi ipsilateral, Contra contralateral

The regenerated RGC axons further extended to the optic chiasm after passing across the lesion site. CTB^+^ axons could be seen growing laterally into the optic nerve (Supplementary Fig. S5). The optic chiasm is a difficult region for the regenerated RGC axons to pass through.^2^ In the optic nerve/chiasm transition zone (OCTZ) and the central region, some regenerated axons showed axonal turn-backs (Fig. 1c, d). Some axons ectopically grew into the ventral hypothalamus and the contralateral normal optic nerve (Fig. 1c, d). Most axons passed through the optic chiasm to the contralateral optic tract, with a few going to the ipsilateral tract (Fig. 1c, d, e). In addition, a large number of axons projected to the contralateral optic tract via the ventral hypothalamus (Fig. 1d, Supplementary Fig. S6a, b (the sham control group)).

Reconnecting the regenerated RGC axons with the visual nuclei in the brain lays the anatomical basis for restoring visual functions. In the CNTF-chitosan group, the regenerated RGC axons projected to the bilateral optic tracts and terminated in the visual nuclei in the brain, including suprachiasmatic nucleus (SCN), medial terminal nucleus (MTN), lateral geniculate nucleus (LGN), olivary pretectal nucleus (OPN) and superior colliculus (SC) (Supplementary Fig. S7, Fig. 1f). The regenerated RGC axons projected to the bilateral visual nuclei, but mainly to the visual nuclei on the contralateral side (Supplementary Fig. S7, Fig. 1f). Whether the axons in the central visual nuclei are nascent or spared should be clearly demonstrated. In the CNTF-chitosan group, GAP43 was highly expressed in the CTB^+^ axon terminals in the SC (Supplementary Fig. S8). Moreover, the nascent axons in the SC were enwrapped by immature oligodendrocytes (Supplementary Fig. S9). In addition, we occasionally found immature projections to the deep layer of the SC (Supplementary Fig. S10a–I). These results suggested that these CTB^+^ axons in the SC were nascent rather than spared. Since the SC was the most distant visual nucleus from the eyeball, it was reasonable to assume that CTB^+^ axons in other visual nuclei were also nascent. The reestablishment of synapses in the LGN is critical in the recovery of depth perception.^3^ In the CNTF-chitosan group, CTB^+^ axon terminals in the LGN were co-labeled with the presynaptic marker vesicle glutamate transporter 2 (Vglut2) and closely adjacent to the postsynaptic density protein 95 (PSD95) (Fig. 1g), suggesting synaptic reorganization. Immunoelectron microscopy further confirmed that CTB^+^ axon terminals (labeled with gold nanoparticles) formed synaptic connections with neuronal dendrites in the LGN (Fig. 1h).

CNTF-chitosan also proved its role in protecting RGCs. In the empty tube group, the number of RGCs was only 9.5% (268.3 ± 44.5 cells/mm^2^) that of the sham control group (2,838 ± 158.3 cells/mm^2^) (Fig. 1i, j). While in the CNTF-chitosan group, the number of surviving RGCs was significantly increased to 30.3% (861 ± 150.7 cells/mm^2^) (Fig. 1i, j).

Seven weeks after optic nerve injury, we injected CTB into the contralateral SC to retrogradely trace the regenerated visual pathway (Supplementary Fig. S11a, b). CTB signals were found across the optic chiasm and the lesion area (Supplementary Fig. S11c, d, Supplementary Fig. S6c, d (the sham control group)). CTB^+^ cells were seen expressing Tuj1 in the ganglion cell layer (Supplementary Fig. S11e, f). some CTB^+^ RGCs expressed melanopsin (Supplementary Fig. S11g), suggesting that these RGCs were M-RGCs.

Restoration of visual functions is the ultimate goal of this study. We used flash visual evoked potential (F-VEP), pupillary light reflex (PLR), dark-light preference (DLP), and visual cliff test to evaluate the recovery of visual functions in rats. First, we measured the F-VEP. 80% of the rats in the CNTF-chitosan group could evoke VEP (Fig. 1k). Compared with the sham control group, the latency of the P1 wave slightly prolonged (71.83 ± 24.05 ms vs. 60.82 ± 14.94 ms), and the amplitude of the N1-P1 wave slightly decreased (2.873 ± 1.437 μV vs. 4.975 ± 2.237 μV) (Fig. 1l, m). Next, we tested the PLR. After dark adaptation, the rats were continuously given light stimulation, and the pupil changes were recorded within one minute. In the CNTF-chitosan group, seven rats had repeated pupil constriction and dilation throughout the recording process (the Marcus Gunn pupil)^4^ (Fig. 1n). At the end of the recordings, these pupils remained moderately constricted (Fig. 1n). Then, we carried out the DLP. In the CNTF-chitosan group, most rats were able to complete the task (Fig. 1o). Finally, we examined the visual cliff test. Rats in the CNTF-chitosan group stayed at the shallow end 82.9% of the time during the entire test, close to that of the sham control group (Fig. 1p).

To sum up, CNTF-chitosan not only induced long-distance regeneration of RGC axons but also partially restored the visual functions. This degree of optic nerve regeneration has rarely been reported before. Long-distance axonal regeneration after CNS injury remains challenging.^5^ This study has confirmed that CNTF-chitosan can successfully repair the adult mammalian visual system, thus pointing out a new direction for regenerating the CNS.

## Supplementary information

Supplementary Information

## Data Availability

All data generated or analyzed during this study are included in this published paper.
