# Supplementary material for: Regeneration and functional recovery of the completely transected optic nerve in adult rats by CNTF-chitosan
Source: Signal Transduct Target Ther. 2023 Feb 27;8:81. doi: 10.1038/s41392-022-01289-0 (PMC9968709; doi:10.1038/s41392-022-01289-0)
Supplement: Supplementary file 1 — Supplementary Information [file 41392_2022_1289_MOESM1_ESM.docx]

Supplementary information

**Regeneration and Functional Recovery of the Completely Transected Optic Nerve in Adult Rats by CNTF-chitosan**

Xiao Liu^1,12^, Fei Hao ^2,12^, Peng Hao^1^, Jingxue Zhang^4^, Liqiang Wang^5^, Si-Wei You^6^, Ningli Wang^4^, Zhaoyang Yang^1^*****, Kwok-Fai So^7,8,9,10,11^*****, Xiaoguang Li^1,2,3,13^*****

^1^Department of Neurobiology, School of Basic Medical Sciences, Capital Medical University, 100069 Beijing, China.

^2^School of Engineering Medicine, Beijing Key Laboratory for Biomaterials and Neural Regeneration, Beihang University, 100083 Beijing, China.

^3^Beijing International Cooperation Bases for Science and Technology on Biomaterials and Neural Regeneration, Beijing Advanced Innovation Center for Big Data-Based Precision Medicine, Beihang University, 100083 Beijing, China.

^4^Beijing Tongren Eye Center, Beijing Tongren Hospital, Capital Medical University, Beijing Ophthalmology and Visual Sciences Key Laboratory, 100005 Beijing, China.

^5^Department of Ophthalmology, The Third Medical Center, Chinese PLA General Hospital, 100089 Beijing, China.

^6^Department of Ophthalmology, Xijing Hospital, The Fourth Military Medical University, 710032 Xi’an, Shanxi Province, China.

^7^Guangdong-Hongkong-Macau Institute of CNS Regeneration, Ministry of Education CNS Regeneration Collaborative Joint Laboratory, Jinan University, 510632 Guangzhou, Guangdong Province, China.

^8^Bioland Laboratory (Guangzhou Regenerative Medicine and Health Guangdong Laboratory), 510530 Guangzhou, Guangdong Province, China.

^9^Department of Ophthalmology and State Key Laboratory of Brain and Cognitive Sciences, The University of Hong Kong, 999077 Hong Kong, China.

^10^Center for Brain Science and Brain-Inspired Intelligence, Guangdong-Hong Kong-Macao Greater Bay Area, 510515 Guangzhou, Guangdong Province, China.

^11^Co-innovation Center of Neuroregeneration, Nantong University, 226001 Nantong, Jiangsu Province, China.

^12^These authors contributed equally.

^13^Lead Contact

*****Correspondence

Email: X.G.L.: [lxgchina@sina.com](mailto:lxgchina@sina.com);

K.F.S.: [hrmaskf@hku.hk](mailto:hrmaskf@hku.hk);

Z.Y.Y.: [wack_lily@163.com](mailto:wack_lily@163.com)

The supplementary information includes:

Extended Discussion

Materials and Methods

Supplementary Figures. S1 to S11

**Extended Discussion**

In this study, we showed clear evidence that CNTF-chitosan provided a favorable microenvironment to promote long-distance axonal regeneration of RGCs. The nascent axons reestablished contact with the visual nuclei in the brain and partially restored the visual functions. Moreover, some nascent RGCs axons, including some from M-RGCs, regenerated over long distances. In summary, the adult mammalian visual system could be partially reconstructed with the help of CNTF-chitosan after severe injury.

Nascent axons need to be strictly distinguished from spared axons after the CNS injury.^1^ According to the criteria of axonal regeneration proposed by previous studies, the nascent axons in our study were systematically demonstrated.^2^ 1. Model selection. The optic nerve crush model was mostly used in previous studies. In this study, the optic nerve transection model was selected. The nascent axons were clearly observed growing out of the proximal stump of the optic nerve (Fig. 1b, Supplementary Fig. S3), and the nascent axons could also be found between the bioactive materials (Fig. 1b, Supplementary Fig. S3). 2. Axonal morphology. Owing to the influence of various factors, the nascent axons grew on irregular trajectories, showing axonal turn-back and axonal bifurcation (Fig. 1b, c, d; Supplementary Fig. S3). 3. Axonal projection. When the nascent axons passed through the optic chiasm, some axons mistakenly projected to the ventral hypothalamus and the contralateral normal optic nerve (Fig. 1c, d). 4. The neogenic properties of the axons in the central visual nuclei. GAP43 was highly expressed in the axon terminals of RGCs in the SC, and these axons were surrounded by immature oligodendrocytes (Supplementary Fig. S8, 9). In the meantime, we observed the immature projections deep in the SC (Supplementary Fig. S10a-I). These aspects strongly confirmed that CNTF-chitosan could induce the axonal regeneration of RGCs over a long-distance and form a new visual pathway.

Previous studies have shown that RGCs axons can grow over long distances in the peripheral nerves after optic nerve transection.^3,4^ However, in the model of optic nerve crush injury, it was extremely difficult for the nascent axons to travel in the distal degenerate nerve and they rarely reached the optic chiasm.^5^ Further studies have shown that myelin-related proteins and chondroitin sulfate proteoglycans (CSPGs) in the CNS microenvironment inhibit axonal regeneration.^6,7^ In this study, we discovered that the growth of nascent RGC axons was soon terminated when they grew into the distal optic nerve stump (Supplementary Fig. S3-V); quite surprisingly, the newly formed axon bundles grew smoothly from the lateral side of the distal degenerate optic nerve instead (Fig. 1b’’-IV--VI). In the optic nerve, the connective septa divide the optic nerve fibers into axonal bundles. These septa are mainly composed of loose connective tissue, glial cells and blood vessels.^8,9^ We have shown previously that the abnormal recrossing retinotectal projections grew along connective tissue bridge after superior colliculus lesions in newborn Syrian hamsters.^10^ In vitro, dorsal root ganglion (DRG) neurites grew along the connective septa in the optic nerve,^11^ which may contain some components that support the growth of neurites. In this study, the lateral ingrowth of RGC axons in the distal degenerate optic nerve might also accomplish through the connective septa.

After optic nerve injury, the survival ability varies among different RGC types. a-RGCs and M1-RGCs have stronger abilities to withstand injury.^12^ a-RGCs is the type in which PTEN knockdown promotes axonal regeneration,^12^ whereas overexpression of SRY-related HMG-box transcription factor 11 (Sox11) promotes axonal regeneration in non-a-RGCs.^13^ These studies suggest that different treatments induce different RGC types to regenerate axons. In this study, we retrogradely labeled the nascent visual system by injecting CTB into the SC, and observed CTB^+^ M-RGCs in the retina (Supplementary Fig. S11g). Although M-RGCs cannot spontaneously regenerate axons after injury,^14^ we demonstrated that, with the continuous support of CNTF, M-RGCs could regenerate axons to the visual nuclei in the brain. Previous studies have shown that M-RGCs are related to non-imaging vision, which projects to the SCN, ventral LGN, intergeniculate leaf (IGL), and OPN, and participates in the regulation of circadian rhythm and the control of PLR.^15,16^ It is worth noting that some M-RGCs have the ability of visual imaging.^17^ How CNTF-chitosan promotes the ectopic axonal regeneration of M-RGCs to the SC as well as its specific functions needs to be further explored in future research.

The mechanism by which CNTF promotes axonal regeneration of RGCs has been elucidated in detail in the previous studies.^18^ In this study, we hypothesized that CNTF protects RGCs and regenerates their axons through PI3K/AKT/mTOR and JAK/STAT3 pathways. In the next step, we will use single cell sequencing and other methods to complete the comparison of the survival-associated signals and axonal regeneration signals in our CNTF-chitosan model with CNTF-overexpression model and other models. We wish to clarify the specific mechanisms and also explore the role of retinal glial cells in axonal regeneration in the near future.

How to effectively realize clinical transformation of basic research results has long been a pressing challenge for all researchers around the world. Previous measures to promote axonal regeneration of RGCs were mostly based on gene editing, which required intervention before optic nerve injury and put patients at the risk of tumor formation.^5,19^ The bioactive materials used in this study are non-toxic, with good biocompatibility and no side effects. The local administration of bioactive materials at the injured optic nerve will not affect the anatomical structure and physiological function of the eyeball, while keeping pace with the clinical need. This study lays a solid foundation for the clinical repair of optic nerve injury.

**Materials and methods**

**Animals**

Adult female Wistar rats (n=69, 250-300 g) were provided by the Experimental Animal Center of Capital Medical University. All the rats were fed in the SPF animal room of the Experimental Animal Center of Capital Medical University, where the temperature was controlled at 25 ℃ ± 2 ℃, with humidity of 45%-65% and automatic light control (12 h light/ 12 h dark). The animals were free to eat and drink. All animal-related experiments were conducted under the National Institutes of Health Laboratory Animal Care and Use Guidelines and were approved by the Animal Management Committee of Capital Medical University. Rats were randomly divided into the CNTF-chitosan group, empty tube control group and sham control group (n=23 for each group). Because the optic nerve retracted into the brain after transection, which affected the later preparation of specimens, the lesion control group was not set up in this study. Previous studies in our laboratory showed that the chitosan tubes containing chitosan particles could not promote axonal regeneration,^20,21^ so in this study, we only chose chitosan tubes without chitosan particles as the empty tube control group.

**Chitosan conduit and CNTF-Chitosan Preparation**

The preparation of the chitosan tube followed the previous method.^20^ Under sterile conditions, 1 g of di(hydroxyethyl) and 1 g of lithium chloride were added to 100 ml of a 2% (wt/vol) acetic acid and 2% (wt/vol) poly-N-acetylglucosamine [derived from 85% (wt/vol) deamidated chitosan (Sigma-Aldrich)] solution to increase its plasticizing capacity. The mixture was stirred continuously for 5 minutes. A glass capillary (1.0 mm in diameter) was thoroughly cleaned, autoclaved, and dried. It was then vertically immersed in the solution, slowly taken out, kept vertical, and naturally dried. This process was repeated until the outer diameter reached 1.2 mm. After that, this glass capillary was immersed in 2% (wt/vol) NaOH solution for 1 h and then in deionized water. The transparent chitosan tube was removed from the capillary, cut to a length of 2.0 mm, thoroughly sterilized by immersion in 75% (vol/vol) alcohol, and rinsed with PBS.

The preparation of CNTF-chitosan was appropriately modified based on the previous method.^20^ Under sterile conditions, 10 mg of 85% deacetylated chitosan particles (Sigma-Aldrich) was dissolved in 10 ml deionized water (PH 7.2) and allowed to swell for 6 h. The mixture was centrifuged for 10 minutes, and the supernatant was removed. The chitosan particles were frozen at -20 ℃ for 24 h and then placed at 4 ℃ for 10 h. CNTF (Sigma-Aldrich) was reconstituted to 100 ug/ml, and 1 ul of CNTF solution (containing 100 ng CNTF) was mixed with the chitosan particles at 4 ℃. After stirring at 4 ℃ for 6 h, the CNTF-chitosan mixture was cooled and dried in a vacuum. CNTF-chitosan was added to a type I collagen solution, stirred for 30 min, dried, and stored at 4 ℃. 1 mg of CNTF-chitosan (10 ng CNTF) was placed in the middle of a 2.0 mm chitosan tube and stored at 4 ℃.

**Release kinetics of CNTF from CNTF-chitosan**

The release kinetics of CNTF was measured according to the previous study.^22^ 1 mg CNTF-chitosan was placed in the DMEM/F-12 medium at 37 ℃, shaken and left standing. At 1, 3, 6, 12 h, 1-12 w after the start of co-culture, five equal volumes of the medium supernatant were collected separately. The content of CNTF was measured using a human CNTF ELISA kit (Abcam, ab264608). The absorbance was read at 450 nm using an ELISA plate reader (Model 680, Bio-RAD, Japan). The amount of CNTF was determined from a calibration curve based on the concentration of CNTF.

**Optic nerve surgery**

The rats were thoroughly anesthetized with ketamine (100 mg/kg) and xylazine (10 mg/kg) intraperitoneally. The posterior pole of the right eyeball and the origin of the optic nerve were exposed through the supratemporal intra-orbital approach. The optic nerve sheath was opened longitudinally, and the optic nerve was completely transected at 1 mm behind the eyeball. A further 1 mm of nerve tissue was resected distally, and then a chitosan tube containing CNTF-chitosan was inserted immediately to connect the two stumps of the optic nerve (12-0, LINGQIAO SUTURE, Ningbo, China). In the empty tube group, all the procedures were the same as above except that the tube bridging the nerve stumps did not contain CNTF-chitosan. In the sham control group, we opened only the optic nerve sheath. Injury to the ophthalmic artery was avoided throughout the procedure.

**CTB tracing**

CTB anterograde tracing. Seven weeks after the optic nerve injury, CTB-555 was injected into the vitreous to label the RGC axons. Ketamine (100 mg/kg) and xylazine (10 mg/kg) were injected intraperitoneally to anesthetize the rats (six from each group). The injection site was located 1 mm posterior to the corneoscleral junction of the right eye, avoiding damage to the blood vessels. 3 μl CTB-555 (1 μg/μl, BrainVTA, Wuhan, China) was injected into the vitreous with a micro-syringe, and then the erythromycin eye ointment was used to block the scleral tear. The rats were fed normally and perfused after 48 hours.

CTB retrograde tracing. Seven weeks after the optic nerve injury, CTB-555 was injected into the left SC to retrograde label the visual system. Ketamine (100 mg/kg) and xylazine (10 mg/kg) were injected intraperitoneally to anesthetize the rats (five from each group). CTB-555 (1 μg/μl, BrainVTA, Wuhan, China) was injected into the left SC with a micro-syringe [AP (anterior-posterior): 6.5 mm, relative to the bregma; ML (medial-lateral): 1.0 mm; DV (dorsal-ventral): 3.0 mm, relative to the cortex]. The injection volume was 1 μl, the injection speed was 100 nl/min, the needle was retained for 5 minutes after the injection, and the needle was withdrawn at the speed of 1 mm/min. The rats were fed normally and perfused after 10 days.

**Immunohistochemistry**

The CTB-traced rats were over-anesthetized and perfused with 0.9% NaCl solution and 4% PFA. The eyeball, optic nerve, and brain were harvested and postfixed in 4% PFA for 6-8 h, then dehydrated in 30% sucrose and stored at 4 ℃. Tissue sections were prepared using a frozen microtome (Leica). The thickness of the optic nerve was 20 μm, and the thickness of the retina and brain was 30 μm. All sections were first incubated overnight at 4 ℃ using the following antibodies: Rabbit anti-MBP (Abcam, 1:200, Cat. No. ab218011), Chicken anti-GFAP (Abcam, 1:1,000, Cat. No. ab4674), Mouse anti-Vglut2 (Synaptic systems, 1:200, Cat. No. 135421), Rabbit anti-PSD95 (Abcam, 1:200, Cat. No. ab18258), Rabbit anti-GAP43 (Abcam, 1:500, Cat. No. ab75810), Mouse anti-O4 (Millipore, 1:50, Cat. No. MAB345), Rabbit anti-βIII-tubulin (Abcam, 1:200, Cat. No. ab18207), and Rabbit anti-Melanopsin (Abcam, 1:500, Cat. No. ab19306). The secondary antibodies were Goat anti-Rabbit Alexa Fluor 488 (ThermoFisher SCIENTIFIC, 1:200, Cat. No. A-11008), Goat anti-Chicken Alexa Fluor 647 (ThermoFisher SCIENTIFIC, 1:200, Cat. No. A-21449), Goat anti-Mouse Alexa Fluor 647 (ThermoFisher SCIENTIFIC, 1:200, Cat. No. A-21235), and Goat anti-Mouse Alexa Fluor 488 (ThermoFisher SCIENTIFIC, 1:200, Cat. No. A-21121), and the sections were incubated at room temperature for 4 h. Nuclei were labeled with DAPI (ThermoFisher SCIENTIFIC, 1:1000, Cat. No. 62248). Immunolabeled tissues were imaged using a laser confocal microscope (Leica TCS SP8). Statistics of CTB^+^ axons: In the ≥8 longitudinal sections of each nerve, axons were counted at fixed distances from the proximal stump to estimate the total number of regenerated axons (mean ± SD).^23^ Statistics of O4-positive cells: 5 sites with dense O4-positive cells were selected from the SC of each rat (six from each group), and the average values (mean ± SD) were taken by the confocal microscopy (63X), and expressed as the number/0.05 mm^2^.

**Whole retinal immunohistochemistry**

The survival of RGCs was assessed following the previous study.^24^ For the immunohistochemical staining of the whole retinal preparations from the right eye (six from each group), the primary antibody was Rabbit anti-βIII-tubulin (Abcam, 1:200, Cat. No. ab18207), and the retina was incubated at 4 ℃ for 40 h. The secondary antibody was Goat anti-Rabbit Alexa Fluor 488 (ThermoFisher SCIENTIFIC, 1:200, Cat. No. A-11008), and the retina was incubated at room temperature for 1 h. The number of RGCs per square millimeter (mean ± SD) was estimated by averaging the confocal images (63X) of 16 points on the retina.

**Electron Microscopy**

This experiment followed the previous method.^20^ The rats (three from each group) were over-anesthetized and perfused with 4% paraformaldehyde plus 2% glutaraldehyde through the heart. The optic nerve was excised and postfixed in 4% paraformaldehyde plus 2% glutaraldehyde at 4 ℃. The lesion site and the distal stump of the optic nerve were immersed in 1% osmium tetroxide for 2 h, then washed with 0.075 M PBS, dehydrated with gradient alcohol and acetone, and finally embedded in epoxy resin. Semithin sections (1 μm) of the fixed specimens were stained with toluidine blue, and the myelin sheath was observed under a light microscope. Ultrathin sections of the tissue specimens were stained with uranyl acetate and lead citrate, and the specific morphology of the myelin sheath and the organelles in the axons were observed under an electron microscope.

**Immune-electron microscopy**

Immunoelectron microscopy specimens were prepared following the previous study.^20,25^ The rats (three from each group) were subjected to CTB anterograde tracing (as described above); 48 hours later, they were over-anesthetized and perfused with 4% paraformaldehyde and 0.075% glutaraldehyde through the heart. The brain was taken out and postfixed in 4% paraformaldehyde and 0.075% glutaraldehyde at 4 ℃ for 6-8 h. The left lateral geniculate nucleus region was cut into 30 μm slices at 0 ℃ using an oscillating microtome. Tissue sections were washed three times with 0.01 M PBS and ice incubated with 0.1% sodium borohydride for 10 minutes. They were incubated overnight at 4 ℃ with Rabbit anti-CTB (Bioss, 1:100, Cat. No. bs-12862R), and then incubated for 2 h at room temperature using goat anti-rabbit secondary antibody conjugated with 0.4 nm nanogold (Nanoprobes, Inc.). After washing three times with PBS, the sections were incubated in 1% glutaraldehyde for 1 h to immobilize the gold particles. The HQ SILVER kit (Nanoprobes, Inc) was used for 8 minutes to enhance the gold particles. The sections were fixed in 2% osmium tetroxide, then dehydrated in ethanol and acetone, and finally flat-embedded in Epon (19% EMBed-812, 36% DDSA, 44% NMA and 1% BDMA; Electron Microscopy Sciences). To assess the distribution of CTB immunogold particles, the ultrathin sections were obtained only from the outermost layer of the embedded tissue. CTB immunogold particles were located at the axon terminals. The ultra-structures were identified according to the criteria described in the previous study.^20^

**Visual evoked potential**

Rats (ten from each group) were dark-adapted for one hour before recording and anesthetized by intraperitoneal injection of ketamine (100 mg/kg) and xylazine (10 mg/kg). The right pupil was dilated with Tropicamide Phenylephrine Eye Drops. The stainless-steel electrode was directly inserted into the subcutaneous part of the rat’s head, and the recording electrode was placed in the left ear; the reference electrode was placed at the midpoint of the line connecting the posterior edges of both eyes, and kept parallel to the long axis of the body; the grounding electrode was placed in the subcutaneous part of the tail. Each electrode was connected to a multichannel amplifier. When examining the right eye, a black eye mask was used to completely cover the left eye. The experiment was recorded by the ROLAND RETI-scan system, and the impedance of each electrode was ensured to be 2--10 kΩ. The stimulation intensity was 5 db, the stimulation frequency was 1.6 Hz, the passband was 0.5--50 Hz, and the number of stimulations was 100. Each eye was recorded 3 times in total, and each recording was followed by a complete dark adaptation for 10 minutes. The latency of the P1 wave and the amplitude of the N1-P1 wave were measured by the F-VEP system.

**Behavioral tests**

Pupillary light reflex. After 1 hour of dark adaptation, the rats (ten from each group) were sedated by intraperitoneal injection of ketamine (80 mg/kg). A led lamp was placed 15 cm in front of the right eye, and the light intensity was 40 lumen (LM). The changes of the pupils were recorded by a camera. Considering the influence of the melanopsin in the iris on the pupillary light reflex, we adjusted the traditional detection method, that is, to detect the change of the pupil within one minute after the light was given. We sutured the left eyelid and covered the left eyeball with the fingers during the examination to completely block the indirect pupillary light reflex. The rats with reconstructed visual systems showed repeated pupil contraction and dilation during the whole recording process, which indicated a partial recovery of pupil light reflex. The number of rats with pupillary light reflex was statistically analyzed.

Modified dark-light preference test. The detection device was 40 cm long * 50 cm wide * 18 cm high. A hollow opaque triangular prism (the shelter) with a section of an equilateral triangle (side length 12 cm) and a height of 20 cm was placed horizontally in the device. A led lamp (300 LM) was placed above the detection device, and a far-infrared camera was used to record the behavior. Rats were guided by the visual information to drill into the triangular prism. Compared with hiding in dark areas,^4^ this detection method had a higher specificity. Prior to the experiment, the left eyelids of the rats (ten from each group) were completely sutured. Next the rats were allowed to explore the environment freely in the dark for 5 min. Subsequently, the light stimulation was given, and the behavior was recorded for 30 s. Each rat was tested three times in a row and rested for 5 min after each test. Statistical analysis was carried out according to the completion rate.

Visual cliff test. The visual cliff test was based on the previous study.^24^ We made a transparent plexiglass box, 50 cm long * 12 cm wide * 20 cm high. One half of the plexiglass box was placed on a printed black and white checkerboard (squares measured 2 cm * 2 cm), referred to herein as the shallow end. The other half of the box was suspended, and the same checkerboard was placed 100 cm below the plexiglass, here called the deep end. At the beginning of the test, the rats (ten from each group) were placed at the shallow end, and their behaviors were recorded by a camera for 2 min. Each rat was tested 3 times at an interval of 5 min for rest. The mean time at the shallow end was statistically analyzed. Because the bottom plate of the device was horizontal and was cleaned with alcohol after each test, the rats needed to rely on visual information to distinguish between shallow and deep ends.

**Statistical Analysis**

GraphPad Prism 7.0 software was used for statistical analysis. Data are presented as mean ± SD. Normality analysis of the data was performed using the Shapiro-Wilk test. Levene’s test was used to test for homogeneity of variance. One-way ANOVA and Kruskal-Wallis (three groups), Student’s t-test, and Mann-Whitney U test (two groups) were used to determine the statistical differences. Chi-square test was used to compare the two rates. P＜0.05 was considered statistically significant.

**REFERENCES**

1. Fischer, D., Harvey, A. R., Pernet, V., Lemmon, V. P. & Park, K. K. Optic nerve regeneration in mammals: Regenerated or spared axons. *Experimental neurology*. **296**, 83-88 (2017).

2. Tuszynski, M. H. & Steward, O. Concepts and methods for the study of axonal regeneration in the CNS. *Neuron*. **74**, 777-791 (2012).

3. So, K. F. & Aguayo, A. J. Lengthy regrowth of cut axons from ganglion cells after peripheral nerve transplantation into the retina of adult rats. *Brain research*. **328**, 349-354 (1985).

4. You, S. W. et al. Large-scale reconstitution of a retina-to-brain pathway in adult rats using gene therapy and bridging grafts: An anatomical and behavioral analysis. *Experimental neurology*. **279**, 197-211 (2016).

5. Sun, F. et al. Sustained axon regeneration induced by co-deletion of PTEN and SOCS3. *Nature*. **480**, 372-375 (2011).

6. Schwab, M. E. Nogo and axon regeneration. *Current opinion in neurobiology*. **14**, 118-124 (2004).

7. Yiu, G. & He, Z. Glial inhibition of CNS axon regeneration. *Nature reviews. Neuroscience*. **7**, 617-627 (2006).

8. Sawaguchi, S. et al. The collagen fibrillar network in the human pial septa. *Current eye research*. **13**, 819-824 (1994).

9. Fujita, Y., Imagawa, T. & Uehara, M. Comparative study of the lamina cribrosa and the pial septa in the vertebrate optic nerve and their relationship to the myelinated axons. *Tissue & cell*. **32**, 293-301 (2000).

10. So, K. F. Development of abnormal recrossing retinotectal projections after superior colliculus lesions in newborn Syrian hamsters. *The Journal of comparative neurology*. **186**, 241-257 (1979).

11. Sun, J. H. et al. Decellularization optimizes the inhibitory microenvironment of the optic nerve to support neurite growth. *Biomaterials*. **258**, 120289 (2020).

12. Duan, X. et al. Subtype-specific regeneration of retinal ganglion cells following axotomy: effects of osteopontin and mTOR signaling. *Neuron*. **85**, 1244-1256 (2015).

13. Norsworthy, M. W. et al. Sox11 Expression Promotes Regeneration of Some Retinal Ganglion Cell Types but Kills Others. *Neuron*. **94**, 1112-1120.e4 (2017).

14. Li, S. et al. Promoting axon regeneration in the adult CNS by modulation of the melanopsin/GPCR signaling. *Proceedings of the National Academy of Sciences of the United States of America*. **113**, 1937-1942 (2016).

15. Hattar, S., Liao, H. W., Takao, M., Berson, D. M. & Yau, K. W. Melanopsin-containing retinal ganglion cells: architecture, projections, and intrinsic photosensitivity. *Science*. **295**, 1065-1070 (2002).

16. Lim, J. H. et al. Neural activity promotes long-distance, target-specific regeneration of adult retinal axons. *Nature neuroscience*. **19**, 1073-1084 (2016).

17. Stabio, M. E. et al. The M5 Cell: A Color-Opponent Intrinsically Photosensitive Retinal Ganglion Cell. *Neuron*. **97**, 251 (2018).

18. Fischer, D. & Leibinger, M. Promoting optic nerve regeneration. *Progress in retinal and eye research*. **31**, 688-701 (2012).

19. Magri, L. & Galli, R. mTOR signaling in neural stem cells: from basic biology to disease. *Cellular and molecular life sciences : CMLS*. **70**, 2887-2898 (2013).

20. Yang, Z. et al. NT3-chitosan elicits robust endogenous neurogenesis to enable functional recovery after spinal cord injury. *Proceedings of the National Academy of Sciences of the United States of America*. **112**, 13354-13359 (2015).

21. Rao, J. S. et al. NT3-chitosan enables de novo regeneration and functional recovery in monkeys after spinal cord injury. *Proceedings of the National Academy of Sciences of the United States of America*. **115**, E5595-5595E5604 (2018).

22. Yang, Z., Duan, H., Mo, L., Qiao, H. & Li, X. The effect of the dosage of NT-3/chitosan carriers on the proliferation and differentiation of neural stem cells. *Biomaterials*. **31**, 4846-4854 (2010).

23. Kurimoto, T. et al. Long-distance axon regeneration in the mature optic nerve: contributions of oncomodulin, cAMP, and pten gene deletion. *The Journal of neuroscience : the official journal of the Society for Neuroscience*. **30**, 15654-15663 (2010).

24. de Lima, S. et al. Full-length axon regeneration in the adult mouse optic nerve and partial recovery of simple visual behaviors. *Proceedings of the National Academy of Sciences of the United States of America*. **109**, 9149-9154 (2012).

25. Hao, P. et al. Neural repair by NT3-chitosan via enhancement of endogenous neurogenesis after adult focal aspiration brain injury. *Biomaterials*. **140**, 88-102 (2017).

**Supplementary Figures**


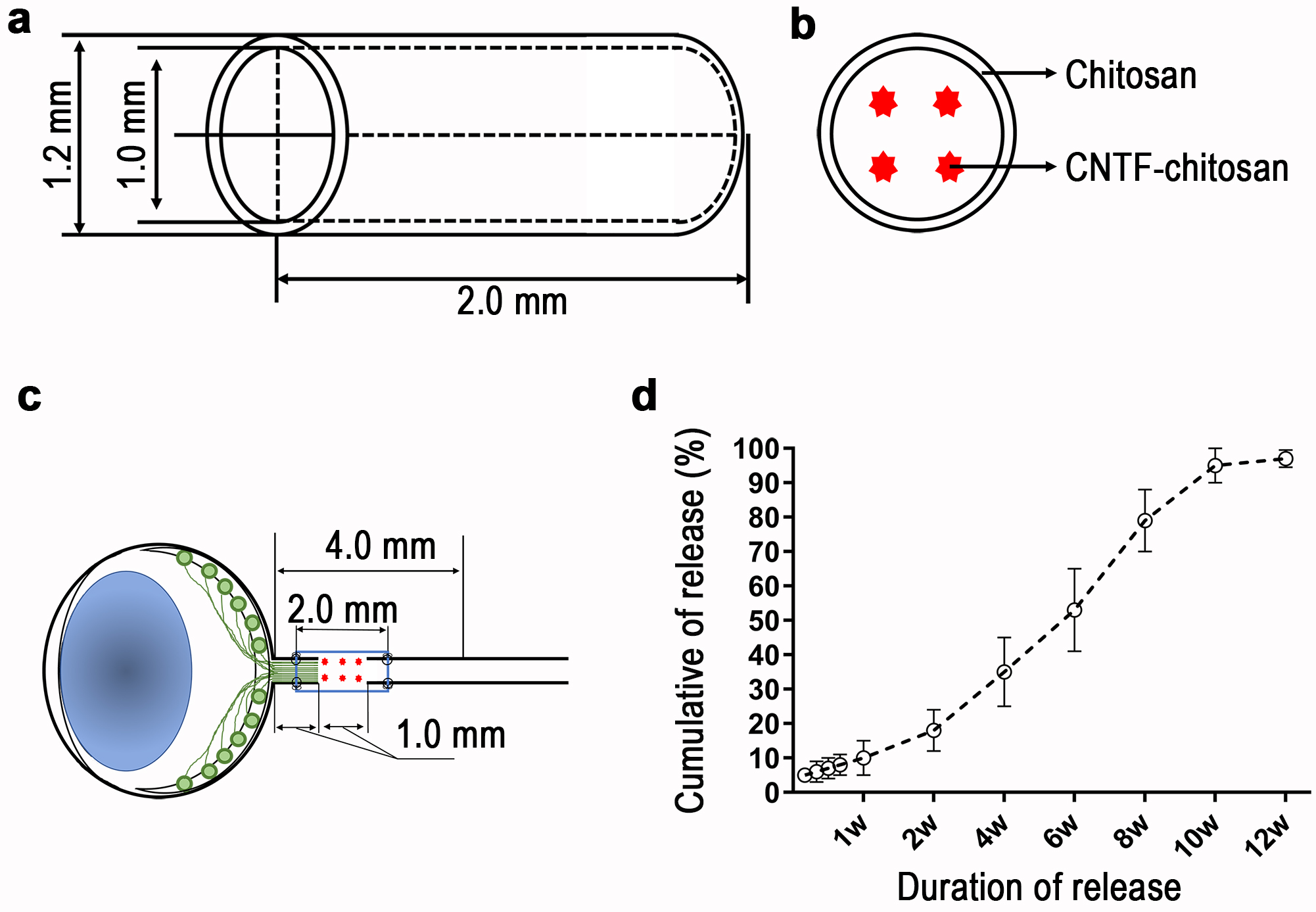


**Supplementary Figure. S1 Schematic diagram of the optic nerve surgery and the release kinetics of CNTF.** (a) Schematic diagram and parameters of the chitosan conduit. (b) Schematic of CNTF-chitosan loading. (c) Schematic diagram and parameters of the optic nerve transection and anastomosis. (d) Release kinetics of CNTF from CNTF-chitosan.


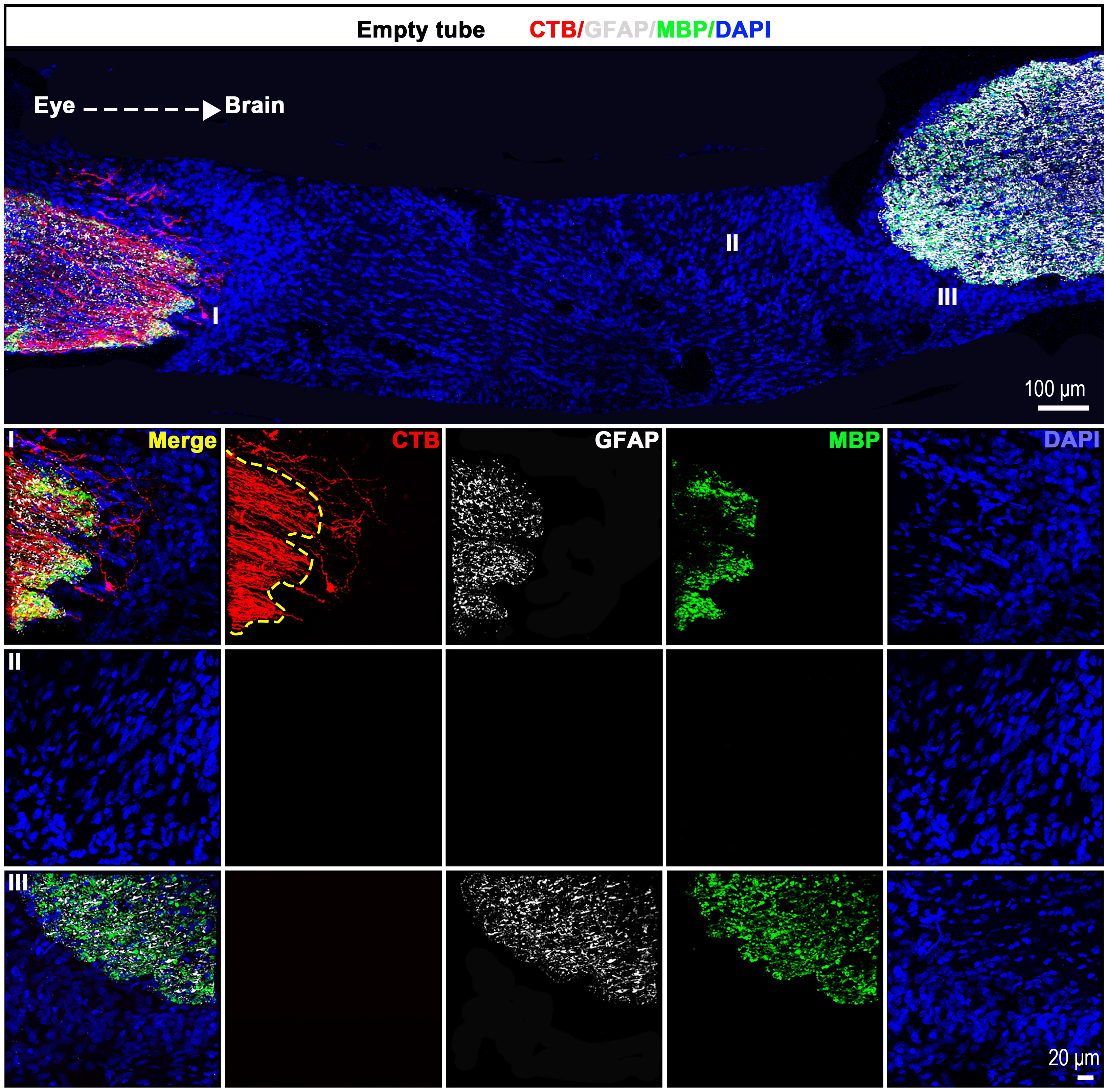


**Supplementary Figure. S2 CTB-labeled RGC axons from the empty tube group.** High-magnification images of the marked regions are shown in (I-III). The yellow dashed lines indicate the boundary of the proximal optic nerve stump.


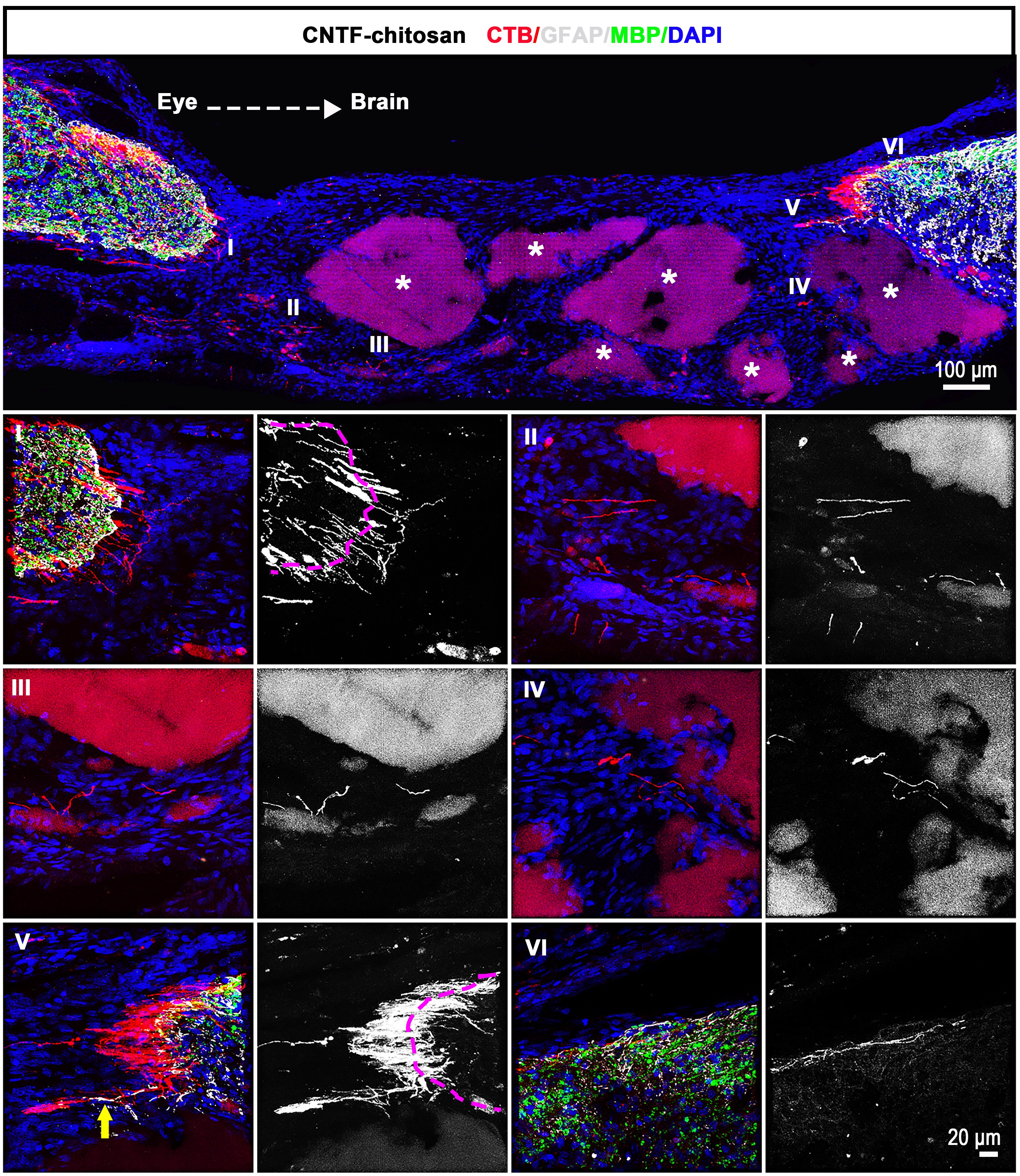


**Supplementary Figure. S3 CNTF-chitosan promotes axonal regeneration of RGCs.** CTB-labeled RGC axons cross the lesion area. High-magnification images of the marked regions are shown in (I-VI). The black and white images are the CTB decolorization images. The dotted pink line indicates the optic nerve boundary. The yellow arrow in (V) indicates axonal growth along the processes of astrocytes. The white asterisks indicate CNTF-chitosan.


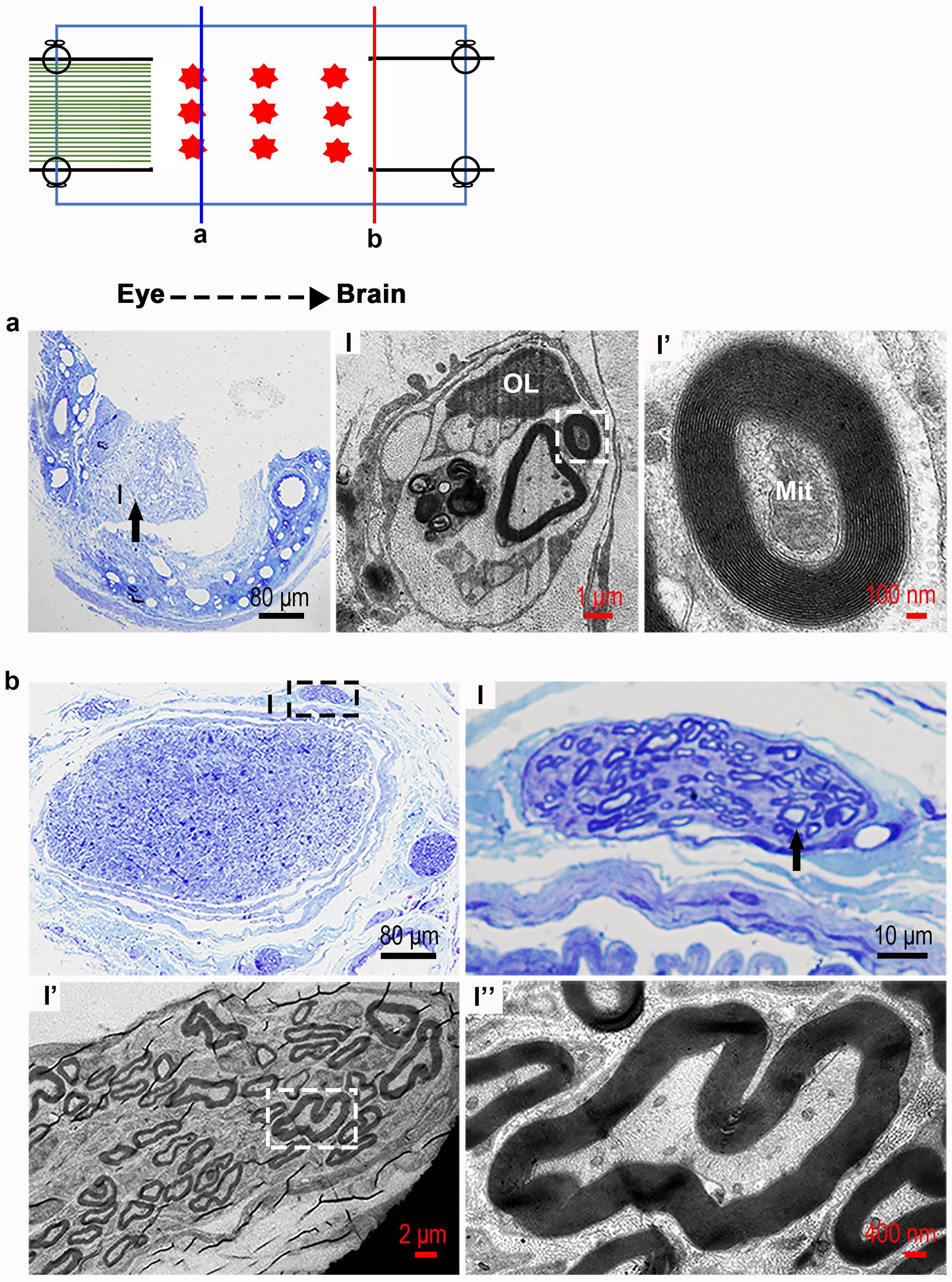


**Supplementary Figure. S4 Toluidine blue staining and transmission electron microscopy of the lesion area.** The upper part is the schematics of the sampling site. (a) Toluidine blue staining of cross section at the middle part of the lesion site (semi-thin section). The transmission electron microscopic image of the black arrowed area is shown in (I, I’; ultra-thin section). OL: oligodendrocyte; Mit: mitochondria. (b) Toluidine blue staining of cross section at the distal optic nerve stump (semi-thin section). The magnified image of the black-boxed region is shown in (I). The transmission electron microscopic image is shown in (I’, ultra-thin section), and a partial enlargement of (I’) is shown in (I’’). The black arrow in (I) indicates a trapezoidal axon, and (I’) is a butterfly-shaped axon.


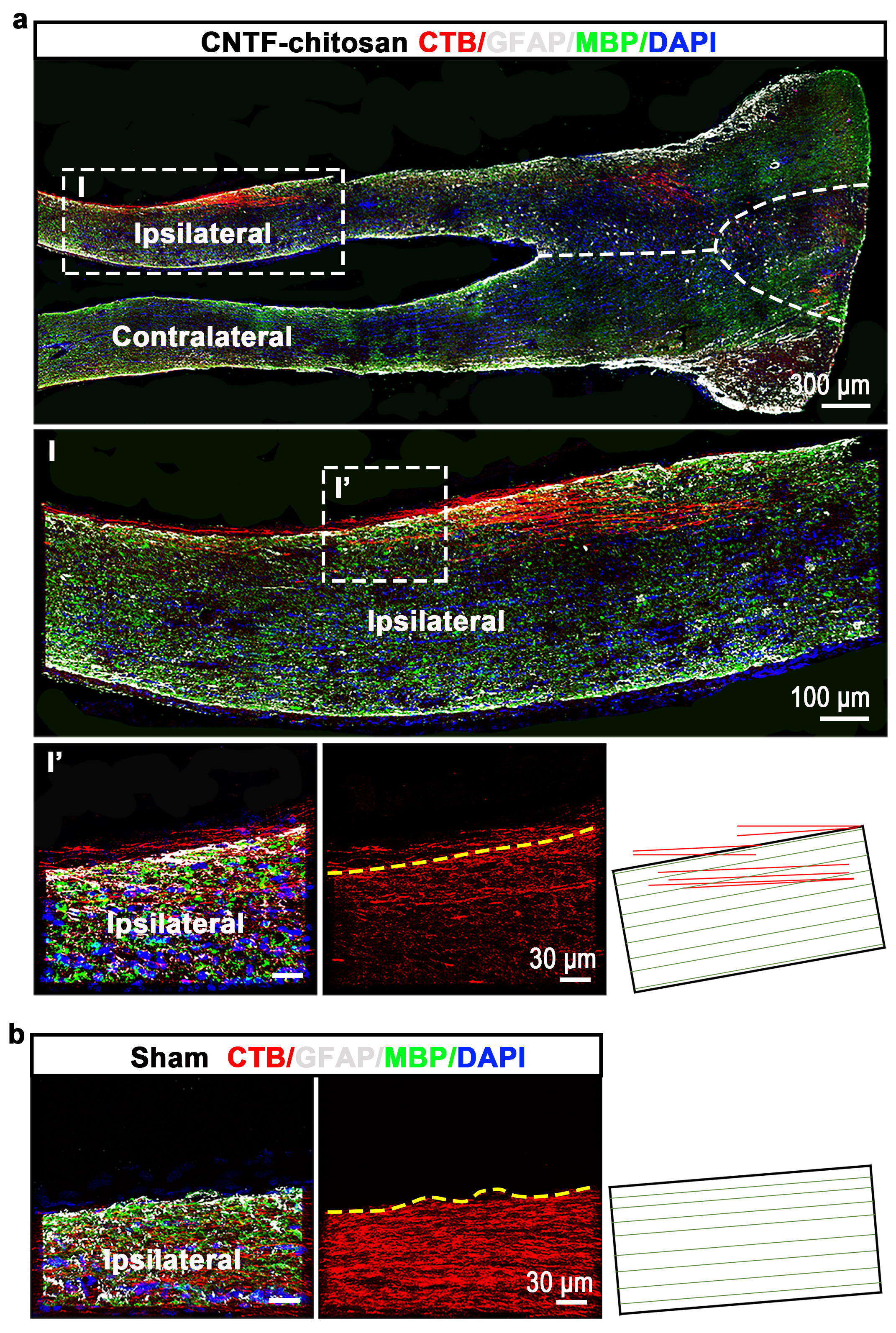


**Supplementary Figure. S5 Long-distance RGC axonal regeneration to the optic chiasm**. (a) In the CNTF-chitosan group, CTB-labeled RGC axons grew around and gradually into the optic nerve. Successive high-magnification images of the boxed regions are shown in (I) and (I’). The white dotted lines indicate the midline of the optic chiasm and the boundary of the optic tract, and the yellow dotted line indicates the boundary of the optic nerve. At the lower right is a schematic representation of RGC axons ingrowing into the optic nerve. (b) The relationship between the CTB-labeled RGC axons and the optic nerve in the sham control group. The yellow dotted line indicates the boundary of the optic nerve, and the schematics are on the right.


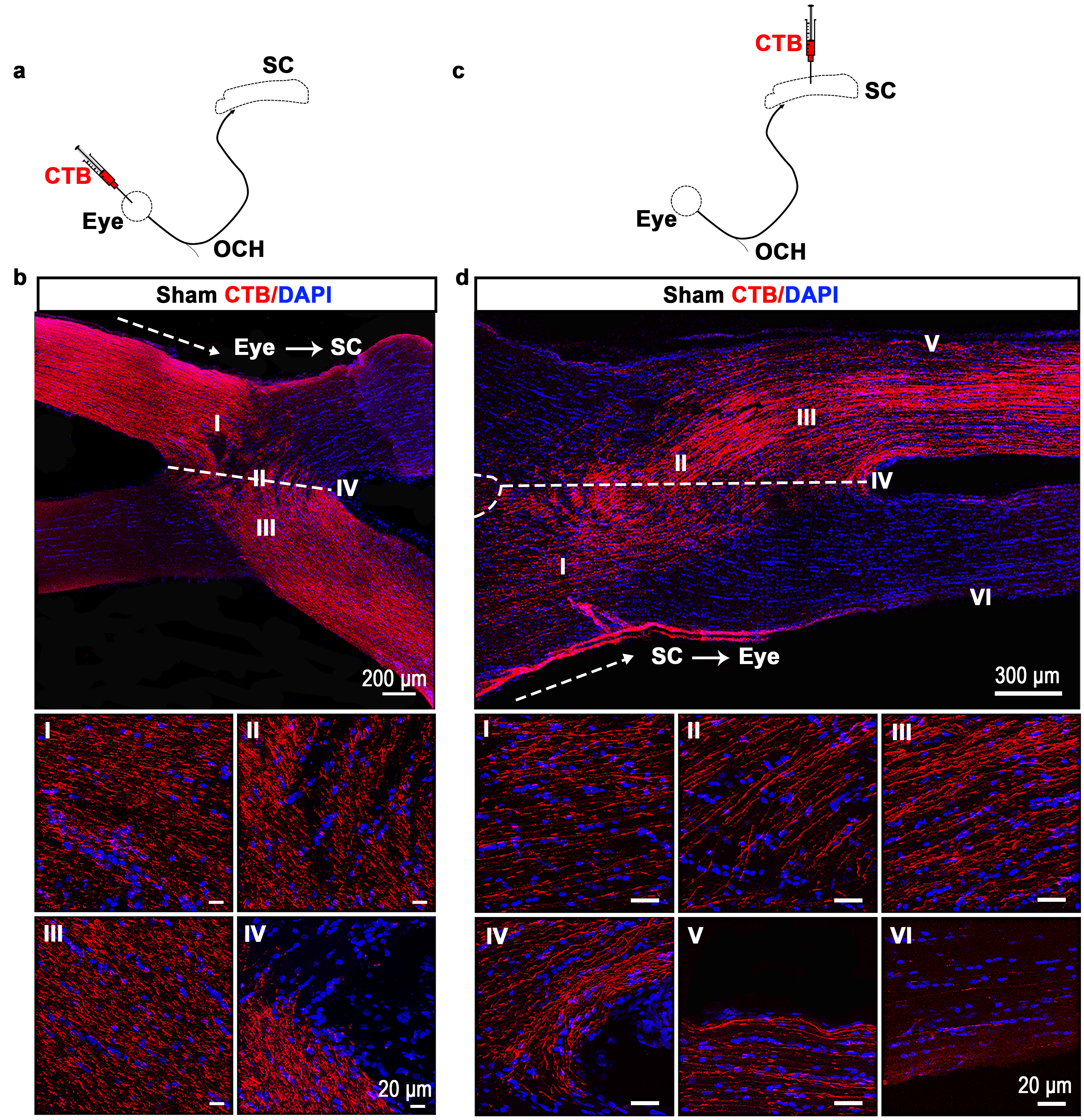


**Supplementary Figure. S6 Anterograde and retrograde tracing of RGC axons in the optic chiasm from the sham control group.** (a) Schematic diagram of visual pathway anterograde tracing. (b) CTB-labeled RGC axons pass anterogradely through the optic chiasm. High-magnification images of the marked regions are shown in (I-IV). (c) Schematic diagram of visual pathway retrograde tracing. (d) CTB-labeled RGC axons pass retrogradely through the optic chiasm. High-magnification images of the marked regions are shown in (I-VI). SC: superior colliculus, OCH: optic chiasm.


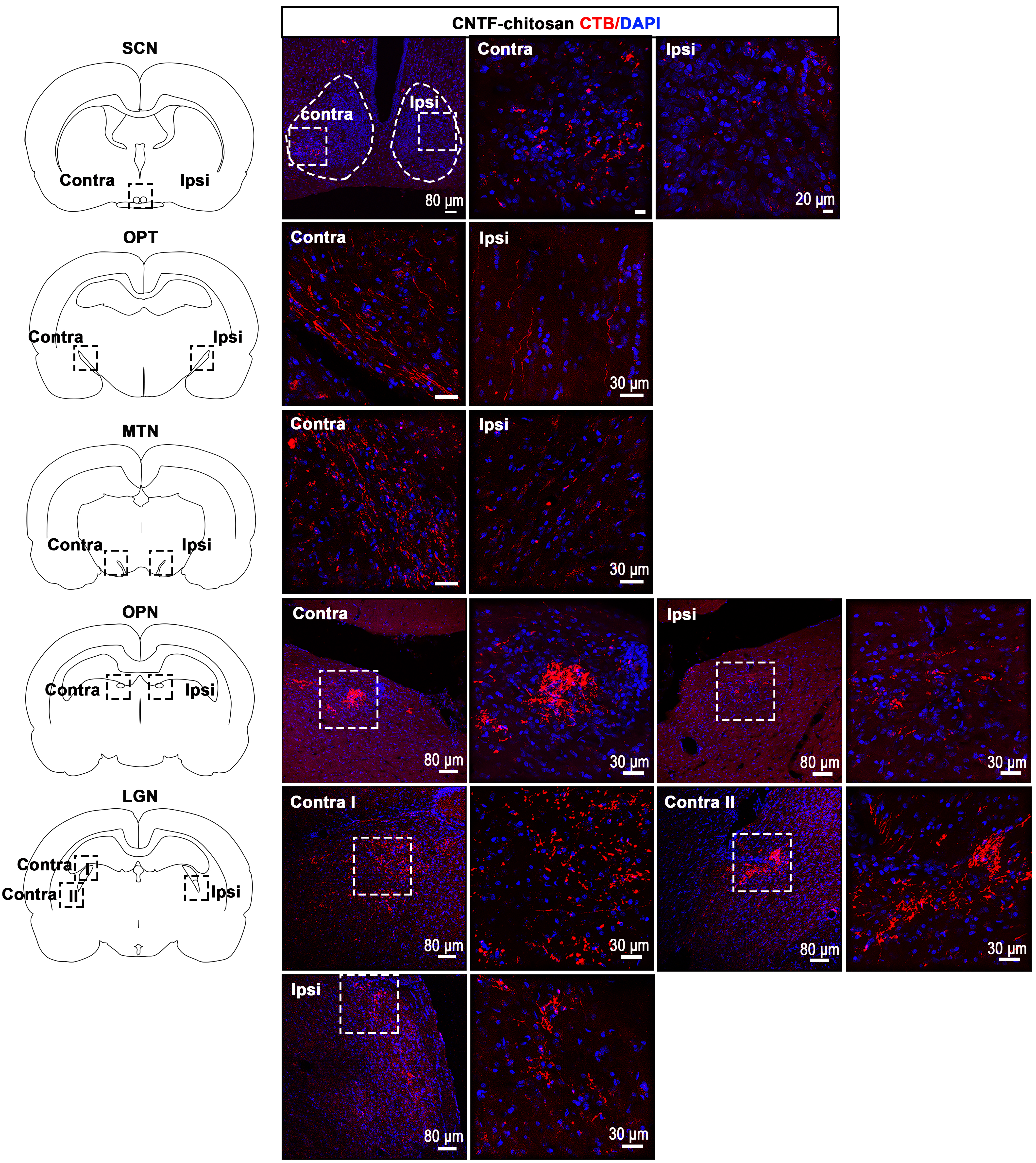


**Supplementary Figure. S7 CNTF-chitosan promotes axonal regeneration of RGCs into the brain.** Vision-related regions in the brain include SCN, OPT, MTN, OPN, and LGN.


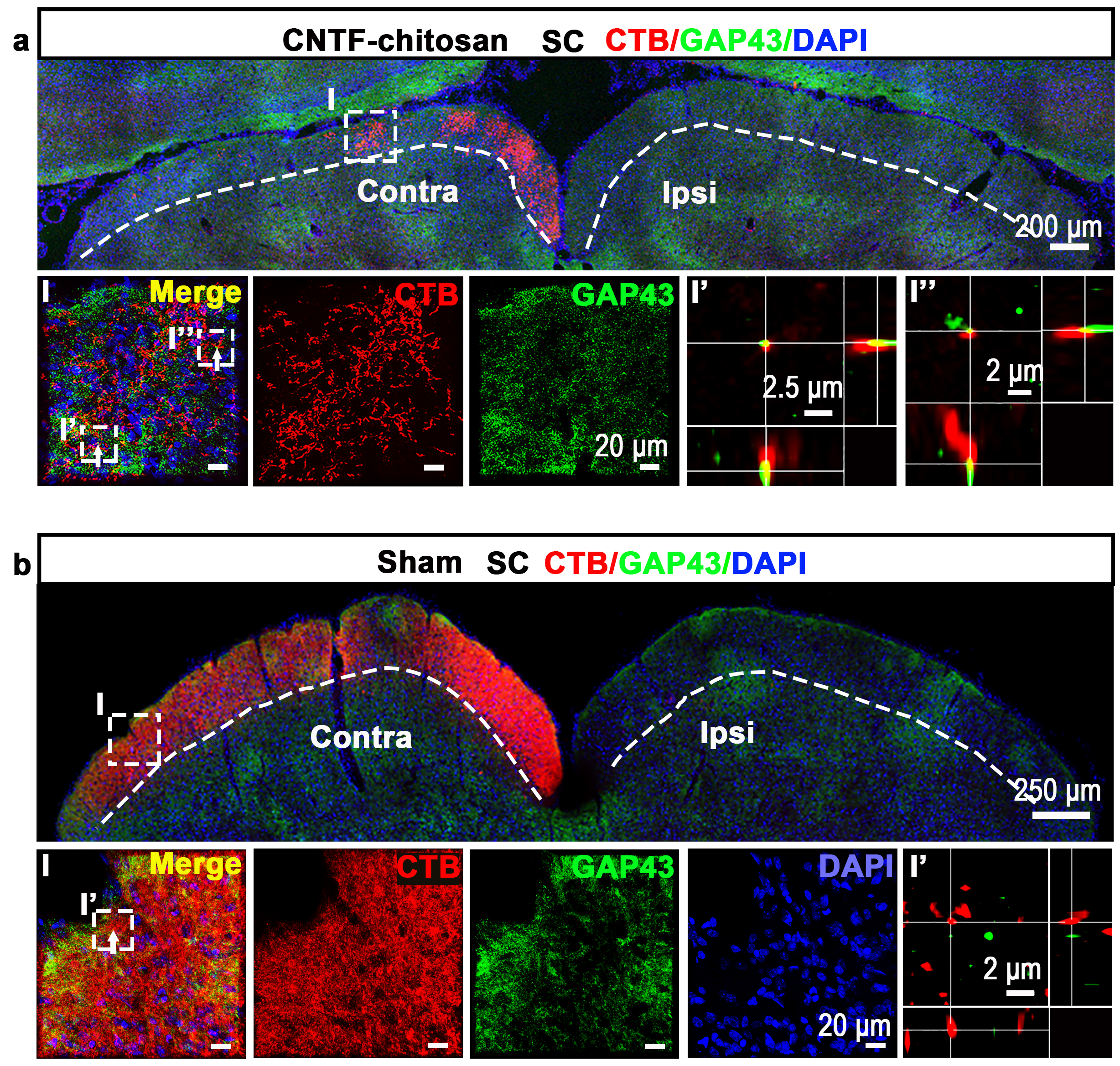


**Supplementary Figure. S8 Neogenic properties of the CTB^+^ axons in the SC.** (a) CTB-labeled RGC axon terminals in the SC of the CNTF-chitosan group express growth associated protein-43 (GAP43). The Z-stacks of the white arrowed areas demonstrate co-labeling. A growth cone-like structure is shown in (I’’). (b) CTB-labeled axon terminals in the SC of the sham control group did not express GAP43.


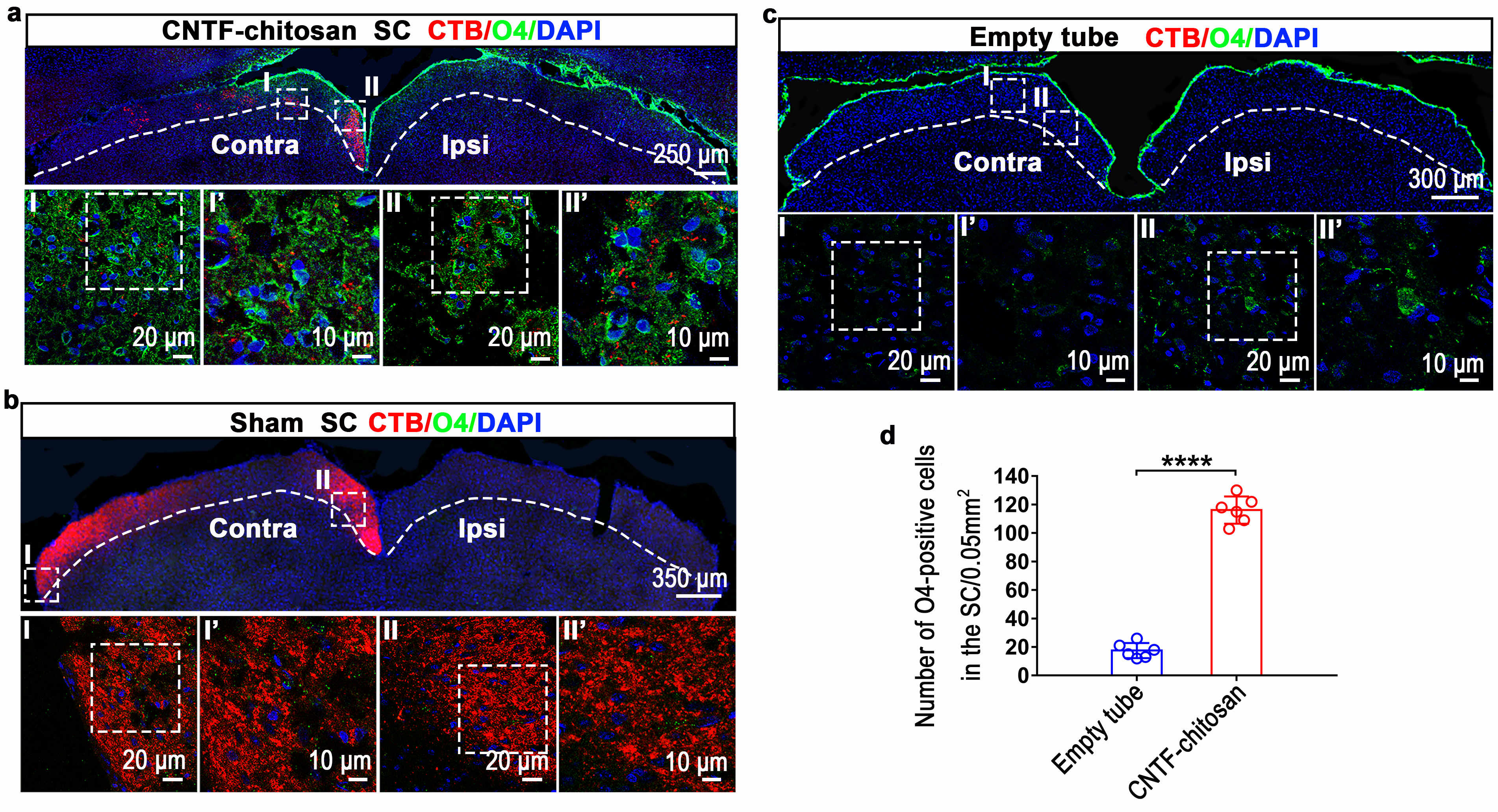


**Supplementary Figure. S9 Neogenic properties of the myelin sheaths in the SC.** (a) CTB-labeled RGC axons in the SC of the CNTF-chitosan group are surrounded by immature oligodendrocytes. (b) No immature oligodendrocytes were found around the CTB-labeled axons in the SC of the sham control group. (c) Few immature oligodendrocytes were observed in the SC of the empty tube group. (d) The statistical analysis of O4-positive cells in the SC. (n=6, mean ± SD, *****P*＜0.0001, unpaired Two-tailed Student’s t-test).


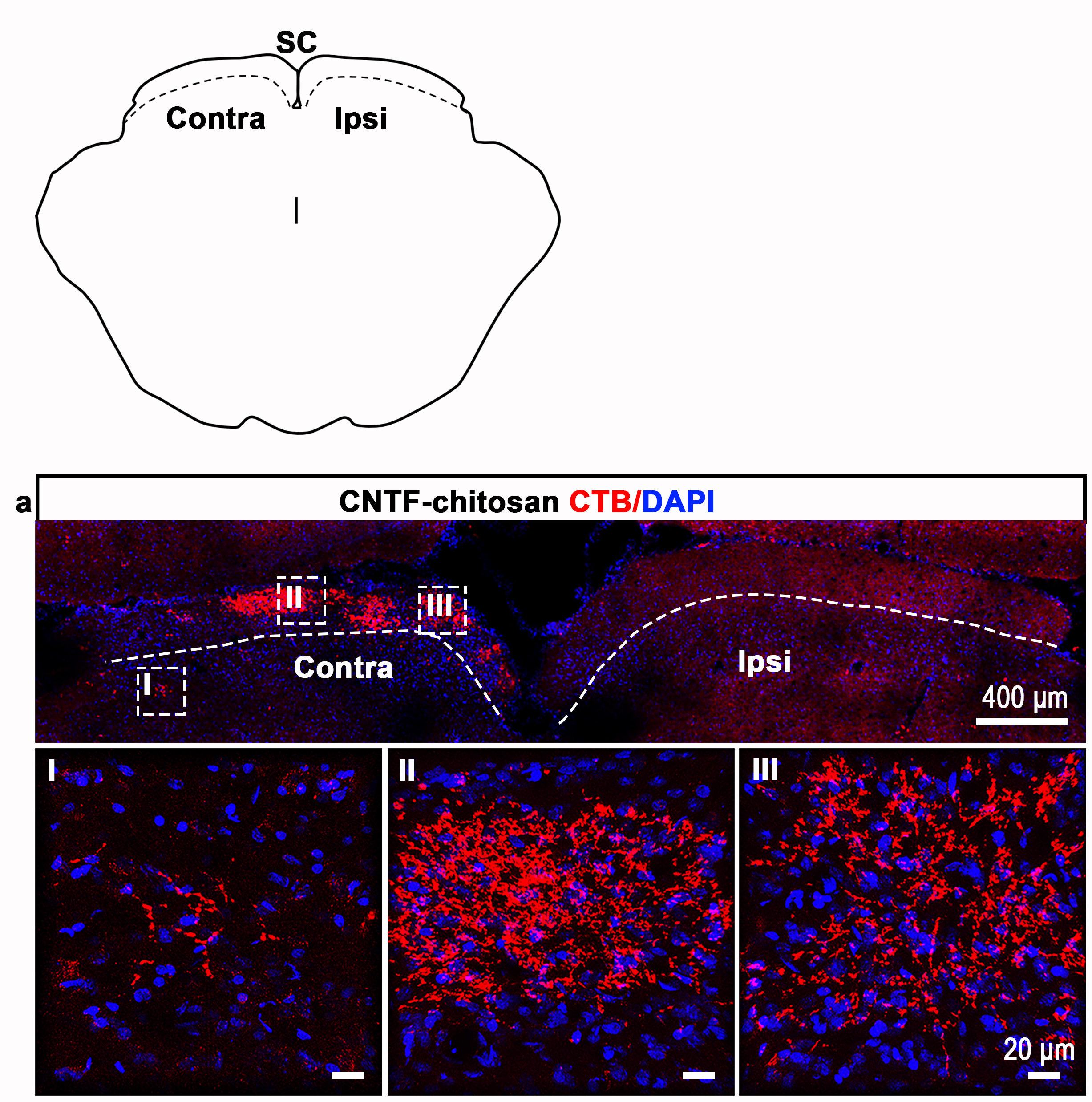


**Supplementary Figure. S10 CNTF-chitosan promotes axonal regeneration of RGCs into the SC.** (a) CTB-labeled axons in the SC. High-magnification images of the marked regions are shown in (I-III). (I) Immature projections in the deep part of the SC.


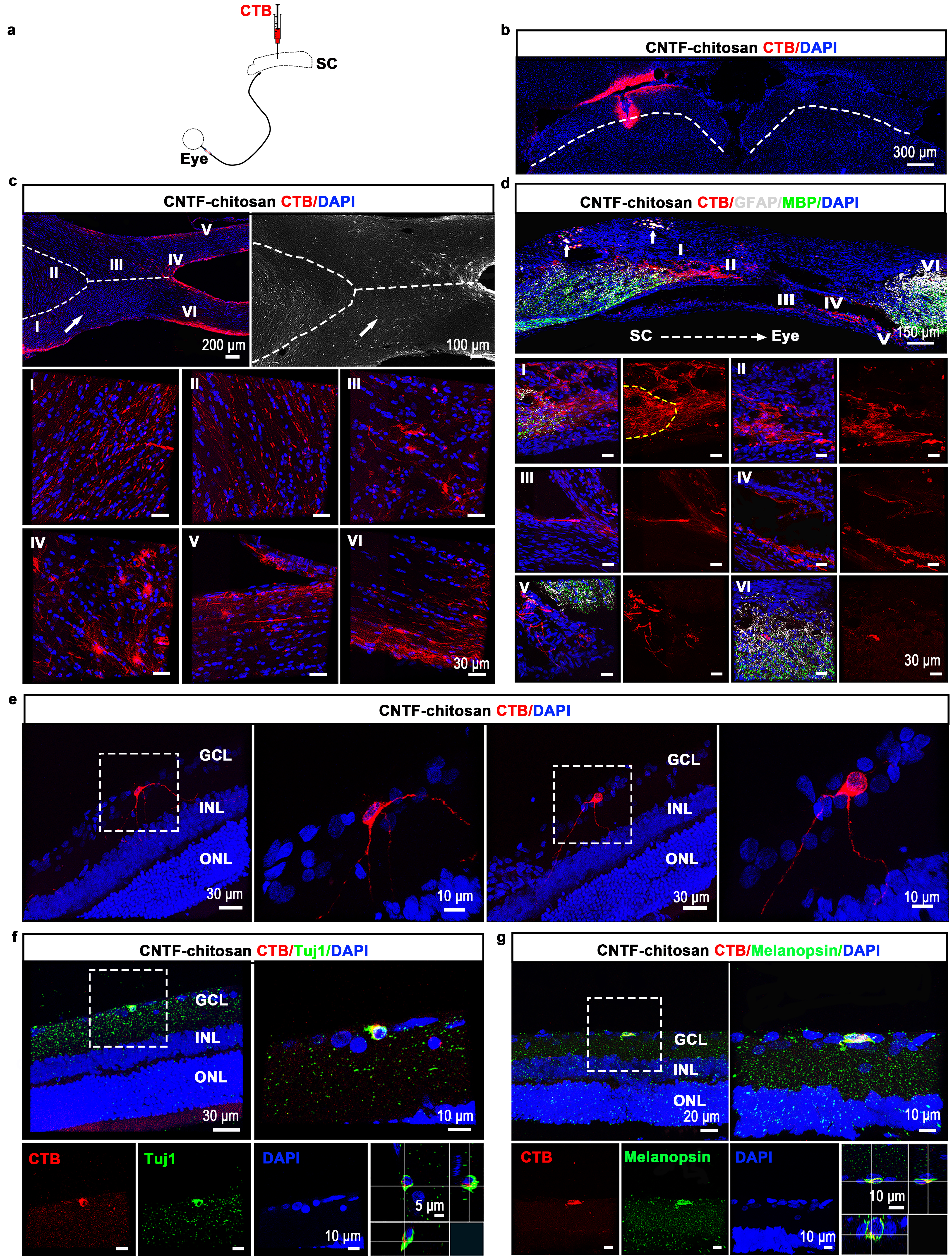


**Supplementary Figure. S11 Retrograde tracing of the nascent visual pathway.** (a) Schematic diagram of CTB retrograde tracing. (b) CTB injection site in the SC. (c) CTB retrogradely passes through the optic chiasm. The black and white image is the decolorized image of the CTB layer. High-magnification images of the marked regions are shown in (I-VI). The white arrow indicates the direction of CTB tracing. (d) CTB retrogradely passes through the lesion area. High-magnification images of the marked regions are shown in (I-VI). The white arrows indicate CNTF-chitosan. (e) CTB-labeled cells in the retinal ganglion cell layer. (f) CTB-labeled cell expresses Tuj1. (g) CTB-labeled cell expresses Melanopsin.
